# An IoT Platform Based on Microservices and Serverless Paradigms for Smart Farming Purposes

**DOI:** 10.3390/s20082418

**Published:** 2020-04-24

**Authors:** Sergio Trilles, Alberto González-Pérez, Joaquín Huerta

**Affiliations:** Institute of New Imaging Technologies, Universitat Jaume I, Av. Vicente Sos Baynat s/n, 12071 Castellón de la Plana, Spain; algonzal@uji.es (A.G.-P.); huerta@uji.es (J.H.)

**Keywords:** Internet of Things, IoT platform, microservices, serverless, smart farming, vineyar diseases

## Abstract

Nowadays, the concept of “Everything is connected to Everything” has spread to reach increasingly diverse scenarios, due to the benefits of constantly being able to know, in real-time, the status of your factory, your city, your health or your smallholding. This wide variety of scenarios creates different challenges such as the heterogeneity of IoT devices, support for large numbers of connected devices, reliable and safe systems, energy efficiency and the possibility of using this system by third-parties in other scenarios. A transversal middleware in all IoT solutions is called an IoT platform. the IoT platform is a piece of software that works like a kind of “glue” to combine platforms and orchestrate capabilities that connect devices, users and applications/services in a “cyber-physical” world. In this way, the IoT platform can help solve the challenges listed above. This paper proposes an IoT agnostic architecture, highlighting the role of the IoT platform, within a broader ecosystem of interconnected tools, aiming at increasing scalability, stability, interoperability and reusability. For that purpose, different paradigms of computing will be used, such as microservices architecture and serverless computing. Additionally, a technological proposal of the architecture, called *SEnviro* Connect, is presented. This proposal is validated in the IoT scenario of smart farming, where five IoT devices (*SEnviro* nodes) have been deployed to improve wine production. A comprehensive performance evaluation is carried out to guarantee a scalable and stable platform.

## 1. Introduction

Over the last decade, the Internet of Things (IoT) paradigm has been applied to a wide variety of scenarios, including smart cities [1], health [2], transportation, industry and agriculture, among others [3,4]. the authors of [5] note that the number of devices connected to the Internet will reach 2.3 trillion by 2025. By connecting billions of devices to the Internet, the IoT has proved its potential to transform society and has attracted attention and funding from academia and industry.

In the case of industry, the IoT has revolutionised the sector producing a substantial economic impact. A vertical form of IoT in the industry sector is Industrial IoT (IIoT), and it is often used in the Industry 4.0 context [6]. IoT is anticipated to be an essential technology in future society [7]; thus, any improvement to it could have a significant economic impact.

The platforms that manage IoT solutions are inherently distributed due to the existence of a vastly diverse range of devices [8]. Such devices carry certain limitations in terms of energy and processing capacity, as well as low storage capacity [9]. the IoT solutions have some general requirements, which can be functional and non-functional [8]. the technical specifications involve managing and discovering IoT devices, handling (acquiring and analysing) received data and generating events/alerts from the IoT data. the non-functional requirements are based on Quality of Service (QoS) and involve scalability, real-time, timeliness, availability and reliability of service, security and reusability.

The race to achieve a universal and generic system of IoT solutions in order to fulfil all these requirements has become one of the pressing scientific concerns of the last few years [10,11,12]. A software ecosystem, traditionally called as middleware (Bandyopadhyay et al. [10]), now baptised as an IoT platform due to its decentralised and mixture pieces, is used to meet all these requirements [13,14]. This component is designed to reduce the complexity of some of the previously mentioned challenges. An IoT platform can help application development by combining heterogeneous devices and encouraging interoperability within the various applications and services working on these devices [15]. It provides a well-defined Application Programming Interface (API) and supports operations which interact with the resources presented by IoT devices or infrastructures.

Currently, there are several IoT platforms in the form of pay-per-service cloud services (Amazon AWS IoT, Azure IoT Suite or DeviceHive, among others). As a requirement, this project is focused on open source software usage to interact with IoT devices. the decision to develop our own IoT platform was due to certain key functionalities of the project, such as a microservices architecture to guarantee the service during the development step and the ability to manage updates on IoT devices. There were not either powerful tools to create custom clients for data visualisation. Moreover, the ability to scale to thousands of connected devices has been considered a key requirement while designing this platform. the present study introduces a complete architecture with which to manage IoT solutions throughout all the layers proposed in [16], with a greater emphasis on the IoT platform stage (or middleware). the suggested platform can efficiently and effectively increase the scalability and management capacity of IoT demands.

More specifically, the contributions of this research study can be summarised as follows. (1) A layered IoT architecture and an IoT platform are proposed with the main objective of providing a centralised and efficient management platform for a wide variety of devices, data and final applications, while meeting all the functional requirements listed above. An example of technological implementation is presented in order to offer a solution, which is reproducible by a third party. (2) This study proposes a solution in which different paradigms in computer science are used, such as microservices and serverless or container-based virtualisation. These features provide an abstraction, thus allowing all previously described non-functional requirements. (3) Validation in a smart scenario is conducted. More specifically, in the area of smart farming, where the goal is improving vine production in vineyard smallholdings. (4) A comprehensive performance evaluation is carried out to guarantee a scalable and stable platform.

The rest of the paper is organised as follows. Section 2 contains the appropriate topics of this paper. Section 3 details the technology-agnostic IoT platform. Section 4 discusses the technological solution used to develop the agnostic approach. Section 5 validates the IoT platform in a smart farming context. A comprehensive performance evaluation is carried out in Section 6. Section 7 compares similar related works. the paper culminates in Section 8 with a discussion as conclusions and recommendations for future work.

## 2. Background

This section details the concepts that will be employed throughout the paper. It defines an IoT lifecycle and proposes an architecture based on this lifecycle. Subsequently, the second section details the microservices concept. Finally, the last section describes the serverless paradigm.

### 2.1. IoT Lifecycle

As noted in the Introduction section, nowadays, there are millions of smart devices using IP-connectivity around the world. Although usually each device has a simple task, such as *Act* or *Observe*, behind them, there are business applications with more ambitious objectives. These business applications configure and deploy IoT devices to follow a strategy in order to reach a final goal.

**Figure 1:**
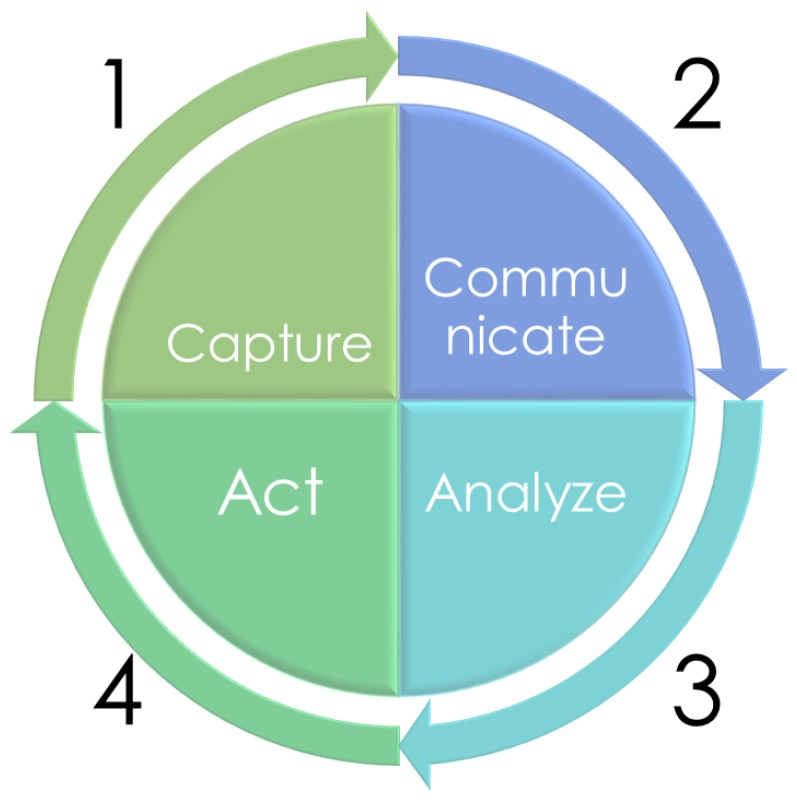
IoT data life cycle: *Capture*, *Communicate*, *Analyse* and *Act*.

These systems can be characterised by following a typical lifecycle in all IoT solutions, that is, to *Capture*, *Communicate*, *Analyse* and *Act* (C2A2) (Figure 1).

The first stage of the cycle, *Capture*, refers to how, through the IoT, devices (hardware) with the observing capability can record a physical phenomenon, such as temperature, ambient light or particulate matter, and transform it into a digital value, which may be quantitative or qualitative.

*Communicate* is the second stage in the lifecycle. In this step, telecommunication technologies are present to link IoT devices with other IoT devices or the central server. Wired or wireless network technologies and protocols defined by the Machine-to-Machine (M2M) communication protocol are used to establish a connection [17].

The third stage, *Analysis*, is responsible for analysing the data that comes from IoT devices. These analyses can be performed in two different ways, batch or in real-time [18,19]. Batch analysis accumulates data in batches and periodically processes each batch. In a real-time analysis, data is analysed at the same time that it is received.

Finally, *Act* is the last stage and implements the business application and, depending on the results obtained in the previous step, it performs specific actions. These “*Acts*” can be of two types: the first, for instance, can be a visualisation of the results from a final user perspective, through a web-client or by sending some notifications provided by analysis. the second can be performed on the same IoT devices with *Act* capability, such as an automatic valve that deactivates a watering function when soil moisture is high.

**Figure 2:**
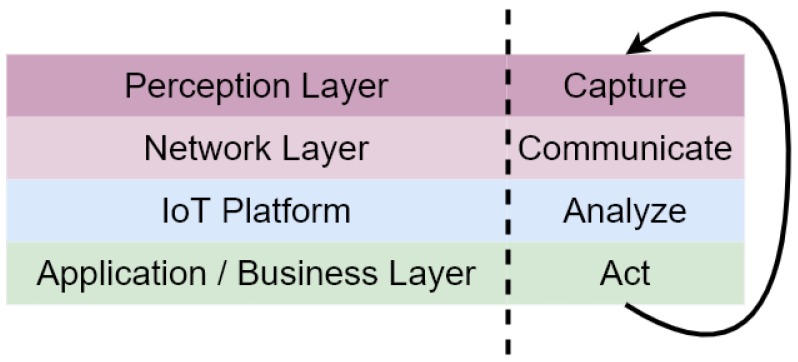
An IoT generic architecture stack.

Figure 2 defines an IoT architecture based on Transmission Control Protocol/Internet Protocol (TCP/IP) [20]. Four layers are described: the Perception layer, the Network layer, the IoT platform layer and the Application/Business layer. Figure 2 equates the defined IoT lifecycle to the IoT architecture. In what follows, a brief description of each layer is provided.

- Perception layer: is at the same level than the physical layer, equivalent to its corresponding in the TCP/IP model. Its purpose is to *Capture* data from each IoT device (state, humidity or temperature, among others).
- Network layer: sends the data from the IoT devices to the upper layer. It can be 2G/3G, Wifi, Zigbee, Bluetooth or Radio Frequency Identification (RFID) technology, among others. It corresponds with the *Communication* stage.
- IoT platform layer: can store and analyse data coming from a IoT device and sends it to be visualised. the IoT platform layer works at the same level as *Analyse* in the IoT lifecycle.
- Application and Business layer: is used in a particular IoT application to manage all the processed data coming from the IoT platform layer, and to apply a business model to a smart scenario, such as smart home, smart agriculture, smart mobility or smart health, among others. It can include graphs, reports, flowcharts, etc. Finally, this layer has the ability to *Act* following the IoT lifecycle.

This IoT architecture is physically located in different sites; in this way, depending on where the computations power is present, we can define three different stages “Cloud”, “Fog” and “Edge” computing. the “Edge” layer includes the IoT devices and users, giving local computing capacity within Things. the second layer, “Fog”, is hierarchical, and aggregates a variable quantity of edge layers. This layer offers other features such as storage, networking, control and data processing. Finally, the “Cloud” layer conducts the final analysis to obtain information and generate knowledge, transferable to decision-making actions [21]. For this work, our approach is to use the cloud computing stage to cover all the IoT lifecycle layers except the first one, perception layer, that will be done at the edge.

### 2.2. Microservices

In a monolithic architecture, all services are developed on a unique repository shared among multiple developers, when a new feature is added or changed they must guarantee that all other services continue working in the same way that they were working. Another inconvenience is when an update is ready to be deployed in the production environment, and all services have to be restarted and thus causing severe issues from the user’s point of view. One final problem, which comes usually with dramatic consequences, is that when one part fails, the entire set of services goes down (Villamizar et al. [22]).

In the context of IoT, where many technologies are integrated, this type of pattern is not recommended [23]. New patterns have been developed to solve these issues. Among them, the microservices pattern, which has scalability and reusability as features [24].

As noted in the first section, one challenge of the proposed IoT platform is to increase scalability and reusability in these kinds of systems. To achieve this, microservices-based architecture is used. This kind of pattern is relatively new [25]. the microservice-based architecture proposes the distribution of the applications in a set of services, in which each one is independent of the others.

This approach allows for the ability to develop, update and scale without interfering with other services. A microservice can be deployed as multiple instances, and different services can be hosted on the same server.

At a high level, a microservice can be represented as a black box, which is divided into layers. These layers are organised depending on the type of task to be performed. the communication between microservices is possible only through the interfaces exposed by the microservice itself. Therefore, each microservice offers an API to facilitate these types of connections.

### 2.3. Serverless

Another paradigm used in the proposed IoT platform is a serverless approach [26]. In general, the term serverless is used to refer to the computing model according to which the provider allows us to execute for a certain period of time portions of code called “functions” without having to take care of the infrastructure. In this model, the provider is in charge of offering the resources transparently, of automatically scaling them if the demand grows and of releasing them when they are not used, defining a series of restrictions on the processing and a payment model for the consumption of the resources used. Serverless architectures are application designs that incorporate *Backend as a Service* (BaaS) third-party services and/or enable the execution of custom code in managed and ephemeral containers provided by *Functions as a Service* (FaaS) platforms. Each of them is described below.

- BaaS: refers to the incorporation of third-party services and applications to manage the logic and status of an application. They are usually applications that use databases in the cloud or third-party services, such as authentication.
- FaaS: allows for the execution of any custom code without provisioning or handling servers.

The main benefits of these types of architectures include the reduction in operating costs, complexity and development time. Additionally, it is considered a sustainable computing solution. Like microservices, they also improve the horizontal scalability of the system [27].

Examples of serverless applications are Amazon Web Services with Lambda (arbitrary code), S3 and DynamoDB (both storage) or Google Cloud with Cloud Functions (arbitrary code), Cloud Firestore (storage) and Cloud Pub/Sub (messaging).

## 3. A Technology-Agnostic IoT Platform

**Figure 3:**
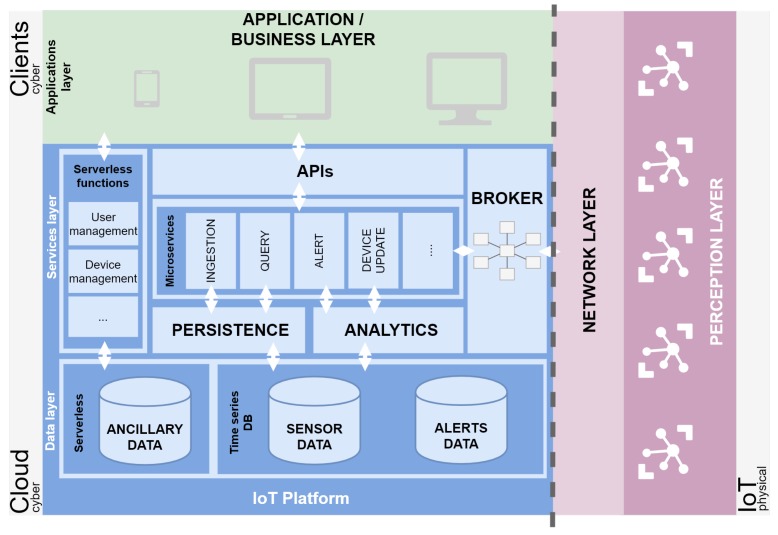
An agnostic-technology IoT architecture.

Although the main objective of this section is to propose an IoT platform to fulfil the requirements defined in Section 1, the generic architecture considers all layers (Figure 3). This architecture is divided in two different scopes: *Physical* and *Cyber*.

The *Physical* scope, on the right side of the dashed line (Figure 3), represents IoT devices from the hardware perspective. Two layers are included in this part: network and perception layers. These two layers cover the IoT devices and, depending on the capabilities of each device, how they can connect to *Capture* or *Act* (Figure 3).

Moving to the left part of the figure, for the *Cyber* scope, two layers are presented in two different environments, *Cloud* and *Client*. the first layer in this scope is the IoT platform itself. the IoT platform is the most important part of the architecture and the key to supporting some important features such as device management, interoperation, security, reusability and the management of huge volumes of data [28].

The IoT platform is formed by different components that we can classify into two distinct layers: Data and Services. the first component is Services, and it is composed of six pieces. These are detailed below.

We start by delineating the service layer. One entry point to the IoT platform is a broker [29]. This component allows connecting with different IoT devices by using multiple protocols. This piece increases the flexibility, scalability and data-level interoperability [30]. It can support some well-known protocols, such as Message Queue Telemetry Transport (MQTT), Advanced Message Queuing Protocol (AMQP) and Simple (or Streaming) Text-Oriented Message Protocol (STOMP). This broker is used as a link to connect the IoT platform-logic with IoT devices. There is a double connection between the two sides, and it can receive data from the edge devices, as well as send data to them.

The broker offers a message queuing system. It provides some benefits such as delivery order and delivery guarantee, redundancy, interface decoupling, flexibility and scalability.

Internally, the IoT platform offers different capabilities or operations to fulfil all functional requirements. These capabilities have been divided into two distinct groups depending on the level of demand and how vital this capability is defined.

This classification defines at a conceptual level how each functionality is implemented. Features that have been identified as most critical, such as the ingestion, will follow the microservices pattern (Section 2.2). All these microservices expose APIs to connect with the other layers.

Following this microservices architecture, four microservices have been designed: *Ingestion*, *Query*, *Alert* and *Device update*. Their number could be expanded depending on more specific solutions. the functionality for each of the microservices is detailed below.

- Ingestion: aims to connect with the Broker to collect data originating from the IoT devices and send this data to the *Persistence* module (described below).
- Query: offers different operations to retrieve historical data provided by the IoT device.
- Alert: is responsible for collecting alerts and propagates them to the final clients.
- Devices: aims to connect to IoT devices in order to send them Over the Air (OTA) updates and ensure the integrity of the IoT devices network.

For the most common IoT platform functionalities such as user and device management, where it is not necessary to have a low-level control of the implementation and the feature is not critical regarding Volume, Velocity and Variety (3Vs), the serverless paradigm is used (Section 2.3).

In this way, two capabilities are defined using this approach:

- User management: manages the registration, removal, discovery and authentication of system users.
- Device management: manages the registration, removal, discovery and authentication of IoT devices.

Another two pieces or modules in the *Services* layer are *Persistence* and *Analytics*. the first module, *Persistence*, is used by some microservices, such as *Ingestion* or *Query*. the primary objective of this module is to store permanent data from the perception layer and other generated data.

These data are stored to recover or apply some analysis. As we previously stated, the *Analytics* module functions to use some analysis, in order to launch specific events/alerts or detect a particular behaviour.

The second layer of the IoT platform is called *Data*, and as its name indicates, it stores all data generated by the system. These data are divided into two categories: data used by microservices and data used by serverless functions. the first category of data is further divided into two groups: observations and alerts data. the first group consists of all the data provided by IoT devices from the perception layer, and a registry of launched alerts. the second group, serverless data, contains the ancillary data needed to carry out the serverless functions.

Moving up to the top of the figure, the IoT platform layer connects with the applications and business layer. This last layer will contain applications to explore all the functionalities provided by the IoT platform and how they will be mixed and used to offer a productive and useful view for an end-user.

## 4. A Technological IoT Full Stack Proposal: *SEnviro Connect*

This section clarifies how the agnostic concepts detailed in Section 3 can be deployed. In this way, the current study proposes an IoT platform (*SEnviro* Connect), as an example of the agnostic-technology architecture. *SEnviro* Connect is an IoT cloud platform capable of managing IoT devices and analysing the data produced from them in order to be useful for a final user.

**Figure 4:**
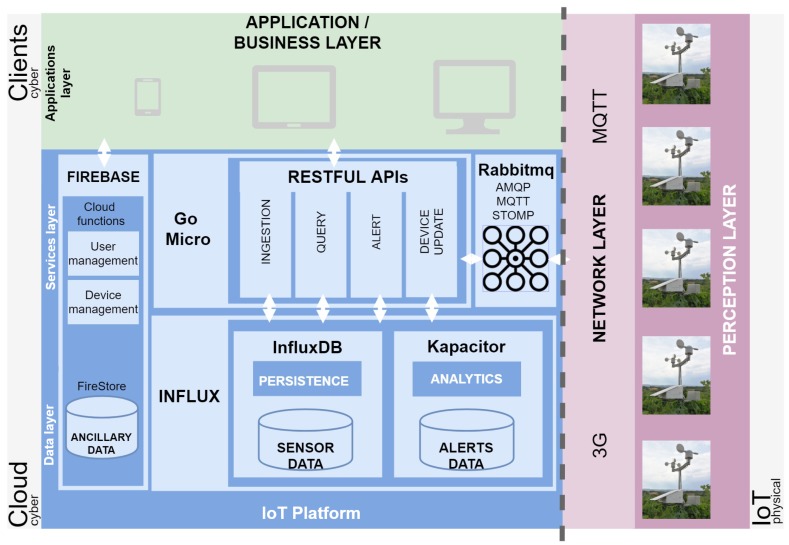
A general overview of the full system from a technological point of view.

Although throughout this study *SEnviro Connect* uses a specific IoT device called *SEnviro* node, it can operate with any IoT device solution. Figure 4 presents a general summary of the full platform from a birds-eye point of view. the dotted line separates *SEnviro* node from *SEnviro* Connect. In what follows, each of these will be detailed.

**Figure 5:**
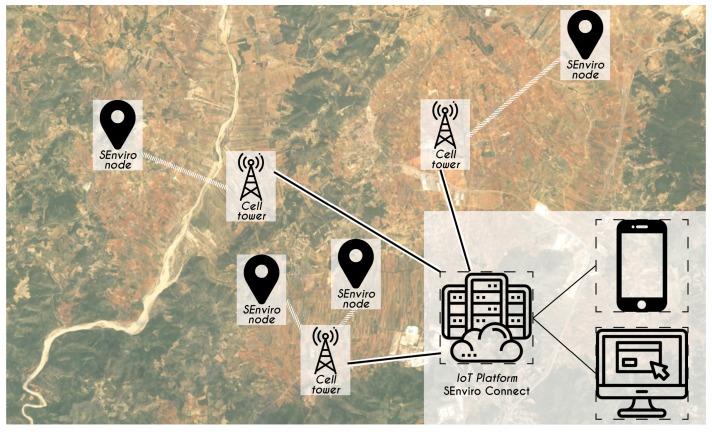
The wireless communication model used to connect devices to IoT platform.

Figure 5 presents the wireless communication model [31], and as we will see in the following sections, different IoT devices will be deployed using 3G connectivity. These devices establish a connection with the IoT platform using cell towers.

### 4.1. SEnviro Node

At the hardware level, the *Perception* layer in Figure 4, a previous design of the *SEnviro* node, has been used. It follows the same elements as the version presented in [32]. In this study, a new version detailed in [33] is used. the main features and improvements from the previous version are listed below.

- The use of a new microcontroller board called Electron. Its design is highly-performant and open source. Electron provides 2G/3G connectivity, which improves the possibility of its installation in any area with mobile data coverage.
- It offers the functionality to receive firmware patches by means of OTA updates. This feature provides a significant increase in terms of improving current software and supporting new functionalities or behaviours without the need to move to the location of each *SEnviro* node.
- It has an improved 3.4 W solar panel with a bigger 2200 mA lithium battery, which, in combination, provides an energy-efficient proposal. To optimise battery life, the *SEnviro* node include energy usage battery-dependent policies in their firmware by default. These policies use the remaining battery level and adapt the frequency of transmissions depending on the battery level and rate of charging.
- Each *SEnviro* node is tagged using a Quick Response (QR) code. It can be used by the time a device has to be added, in order to start receiving data from it. the *SEnviro* node protects all components using a 3D printed case.

On the *Network* layer, each *SEnviro* node includes a 3G module to establish a network connection. From a data transmission point of view, the *SEnviro* node has been developed to follow M2M connectivity. More specifically, this *SEnviro* node supports MQTT connections. This type of connectivity has been chosen because MQTT is an energy-efficient protocol, and the battery is one of its most critical components. the reason for using this protocol is that MQTT uses less power than the Hypertext Transfer Protocol (HTTP) protocol on a 3G network [34,35]. This energy improvement is due to the HTTP protocol overhead, which is more significant than that of MQTT. This aspect is critical in IoT applications where the number of connected IoT devices increases considerably [36].

### 4.2. SEnviro Connect

Moving over to the cyber part, *SEnviro* Connect provides a technological solution to the architecture presented in Section 3. *SEnviro* Connect covers two layers: the IoT platform layer and the Application/Business layer. the last one is tightly coupled to the use case so that it will be detailed in the use case Section 5.

In the cloud part (Figure 4) is where the IoT platform layer resides. Four technologies or tools make up the IoT platform. In the *Services* layer, three different tools have been integrated. These are described below.

- RabbitMQ. This technology has been selected to conduct the broker component. RabbitMQ [37] is an open source message broker and uses a binary application layer protocol, designed to efficiently support a wide variety of messaging applications and communication patterns. It assists in the development of distributed, fault-tolerant and asynchronous applications. From a security point of view, RabbitMQ uses Simple Authentication and Security Layer (SASL) for authentication and data security in Internet protocols. RabbitMQ MQTT offers a wide range of MQTT clients and adds the possibility to interoperate with AMQP and STOMP clients. RabbitMQ operates on top of two core protocol entities. These two entities are called exchanges and queues. Messages published to MQTT topics use a topic exchange internally. Subscribers consume from RabbitMQ queues bound to the topic exchange. These features allow interoperability with different protocols and make it feasible to use a unique management module to examine queue sizes or message rates, among others.
- Micro Mu. Micro Mu [38] is an open source microservices toolkit. It provides a full-stack to build and manage microservices. It has a set of libraries and tools developed in the Go programming language. Micro Mu supplies a single entry point as an API to query microservices, which should be used as the gateway for external access. All *SEnviro* Connect microservices are built using Micro Mu, and an API is provided to connect to via a secure connection using Transport Layer Security (TLS).
- Influx framework. Influx [39] is an open source solution, which provides a set of tools to store and analyse time-series. Two components from Influx have been used. the first one is the InfluxDB. It has a high-performance, efficient database store for handling high volumes of time-series data. It offers a scalable solution through server clustering. It is used as the *Persistence* module to store data provided by *SEnviro* nodes using the ingestion microservice. the second tool is Kapacitor. It can Extract, Transform and Load (ETL) data in order to track arbitrary changes and detect events. Kapacitor can connect in real-time with InfluxDB to get data. It uses a Domain-Specific Language (DSL) named TICKscript to define tasks, which in turn, defines alerts. Each analysis model is encoded using TICKscript; it encompasses the *Analytics* module. All detected events are stored in Firebase, through the use of a specialised microservice. Kapacitor also supports TSL encryption to offer a secure connection.
- Firebase. the last technology that has been used [40]. It is a set of different Google Cloud services: instant messaging, user authentication, real-time database, storage and hosting, among others. As noted above, our *IoT platform* uses serverless services to cover general features with a high level of detail in non-critical capabilities, such as the authentication of the *SEnviro* nodes and users. the Firebase cloud system also provides TSL encryption data transmission.

One of the most notable points of the IoT platform lies in the use of microservices. As described above, microservices have been developed using the Micro-Mu toolkit. Following the microservices detail in Section 3, four different microservices have been developed with specific functionalities (responsibilities): Alert, Ingestion, Query and device microservices. the first microservice is in charge of transmitting the alerts that the platform generates towards the web application. the ingestion microservice is responsible for directing the observations generated by the *SEnviro* nodes towards the database (InfluxDB) for persistence. the third microservice is in charge of consulting the database to retrieve the observations and deliver them to the user. Finally, the device microservice that is responsible for managing (adding and removing) and updating the *SEnviro* nodes on the platform.

These microservices need a services registry to discovery and distribute management services. With that goal, we use Consul [41], which is an open source solution used as service discovery, health checking, key-value store and multi-datacentre. In the current platform is used as service discovery, and the main goal is discovering the address of microservices to establish communication between them. Consul adds the possibility to follow a distributed architecture where service instances have a dynamically assigned network location.

All the tools listed, except Firebase, are wrapped within a container to embed all required dependencies and easily ensure correct configuration. All the proposed architecture can be deployed across different infrastructures, either on a single machine or in a cluster. In this way, this container-based deployment guarantees the scalability and reusability of our approach. Docker [42] is used to achieve this containerisation. Docker images allow to package code and its dependencies in a state known to be stable. This feature enables *SEnviro* connect to be deployed anywhere (in-house server or cloud environment), thus allowing the platform to be up and running from the very first minute after its deployment. Platform components splitted in microservices, each one of them in a separate container image, is another topic worth highlighting. Due to this nature, anybody willing to deploy the platform for their own usage can choose not to deploy certain components unneeded for the use case at hand.

**Figure 6:**
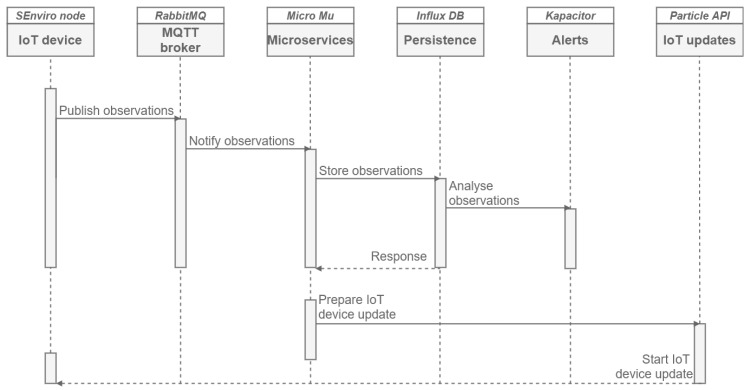
A sequence diagram from the IoT node perspective.

**Figure 7:**
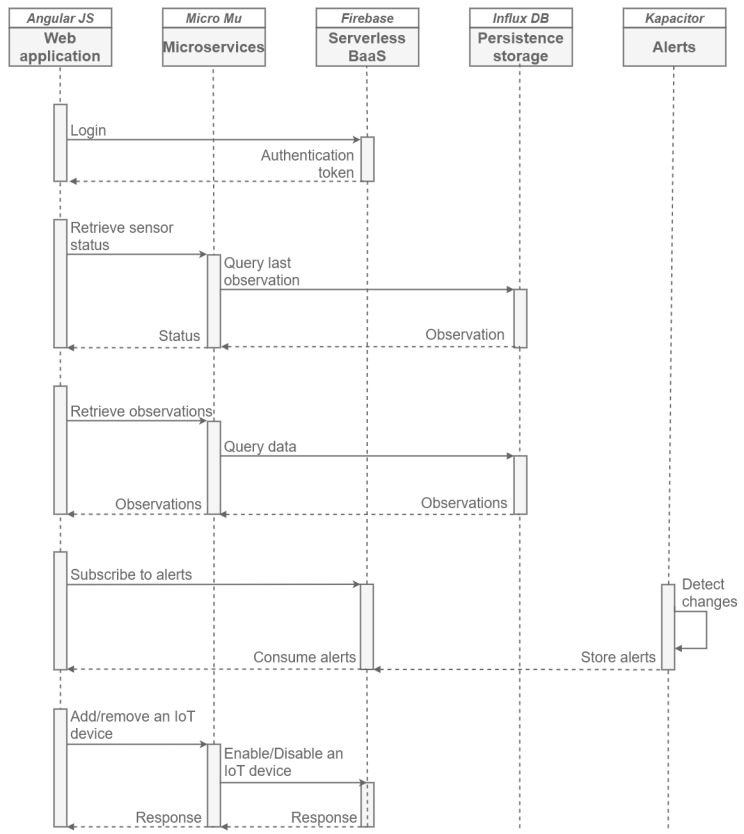
A sequence diagram from the user perspective.

After detailing all IoT platform components, Figure 6 and Figure 7 show two sequence diagrams from an *SEnviro* node (publisher) and a user (consumer) points of view. the first sequence diagram (Figure 6) corresponds to two different scenarios related to the *SEnviro* node. the first scenario enables the publication of observations generated by the *SEnviro* nodes. Each *SEnviro* node sends gathered observations through a previously defined MQTT channel (RabbitMQ). MQTT offers three different levels of Quality of Service (QoS), thus allowing to specify the expected reliability of messages delivery. In this case, our IoT devices use QoS level zero. This level guarantees a best-effort delivery. the QoS level zero does not guarantee the delivery. This level does not incur connection overheads due to the use of User Datagram Protocol (UDP), which works in fire-and-forget basis [30]. In this way, our IoT devices do not expect a response from the broker counterpart to verify if the message has been received or not; moreover, the message is neither stored nor re-transmitted by the sender. This reduces the overall overhead considerably, and thus the total message size. When the broker receives a new observation, the ingestion microservice takes care of the storage and persistence of observations. As previously indicated, InfluxDB has been used to carry out this persistence stage. In turn, Kapacitor is in charge of detecting if there is any change over stored observations, and it performs the pertinent analysis to detect possible problems and thus generate alerts.

The diagram (Figure 6) poses a second scenario to handle *SEnviro* nodes updates. For that purpose, a microservice is in charge of preparing the update and connecting with the API (Particle) that enables the update process and sends the updates to the *SEnviro* nodes.

The second sequence diagram (Figure 7) shows the scenarios from the end-user (web application) point of view, more specifically, five use cases are presented. the first of them is based on an authentication process to enable access to the web application at different levels of privileges (user or administrator). For this, Firebase object is used, which provides these authentication features, providing an access token after verifying the correct authentication. the second scenario details how the user can know the status of the *SEnviro* node in terms of connectivity and battery levels. For this, a microservice capable of reporting the current state has been added. This microservice asks InfluxDB object to retrieve the last observation and obtain the *SEnviro* node status. the third scenario can recover stored observations. the same microservice used to know the status is reused in this scenario. This microservice can recover data from all phenomena with different levels of aggregation from InfluxDB. Another available scene is the ability to detect changes over stored observations and detect possible alerts following defined models. A previous subscription from the web app side is needed to receive the generated alerts; otherwise, the alerts will be stored in Firebase. the last scenario enables the *SEnviro* connect to add and remove IoT devices. For that propose, a microservice capable of interacting with the Firebase object has been designed. In Firebase is stored what IoT devices are registered and which are enabled or disabled.

## 5. Validation in a Viticulture Scenario: *SEnviro* for Agriculture

This section exhibits an example of use in smart farming (called *SEnviro* for Agriculture) to examine and verify the IoT platform presented in the preceding sections. the primary goal of this project is to design and develop a full system for environmental monitoring in agriculture fields. More specifically, it has been applied to the cultivation of the vineyard, with the aim to increase the production quality and yield.

The section is divided as follows. the first subsection shows the context where the *SEnviro* connect has been deployed. the second subsection describes how a IoT devices network is deployed in vineyard fields. Afterwards, a web app to show this kind of data is presented and evaluated.

### 5.1. Viticulture Escenario

Viticulture is a field in which a significant adaptation of IoT platforms has been performed. It has historically been characterised by the high-quality product that can be obtained. Wine growing can be affected by several factors: differences between varietal wines, failing at selecting climatic zones and/or suitable soils, and doubtful winegrower practices in managing the vineyards [43].

*SEnviro* for agriculture is applied for monitoring and discovering vineyard attacks. Based on some previous studies on different models to predict vineyard diseases [44,45,46,47], four different diseases to predict are supported, these are *Black rot*, *Botrytis*, *Powdery mildew* or *Downy mildew*. All these diseases are contingent on certain environmental conditions. An objective of this project is to accommodate the IoT platform to follow these models. the results of integrating these models into the *SEnviro* connect are described and validated in [48].

### 5.2. SEnviro Nodes Deployment

**Figure 8:**
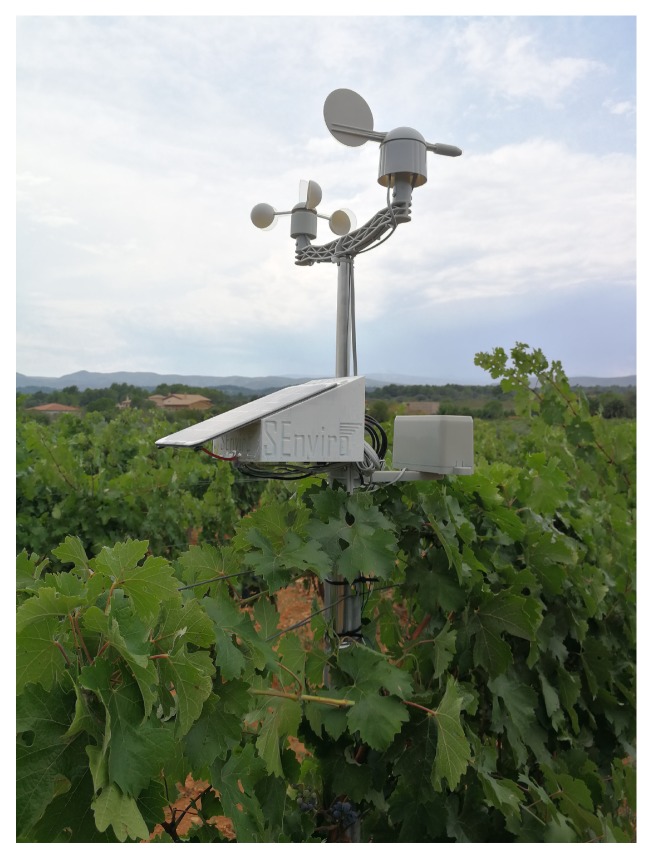
An example of *SEnviro* node deployed on a smallholding.

In this project, five replicas of the *SEnviro* node have been created (Figure 8); four of them have been installed in vineyard fields in Spain, more concretely in a province of Spain, called Castelló, and one unit was reserved for testing proposes in a more accessible and closer location (Table 1). the *SEnviro* nodes have been continuously running for 140 days during the vine season of 2018. Each IoT device has published an updated observation value for each supported observation type every ten minutes during the previously described period.

Some alerts have been generated during this stage, both related the node itself and vineyard diseases. This study only puts forward a validation to test the presented IoT platform and how it can be adapted to support disease models and predict disease in vineyards.

### 5.3. SEnviro Connect Webapp

**Figure 9:**
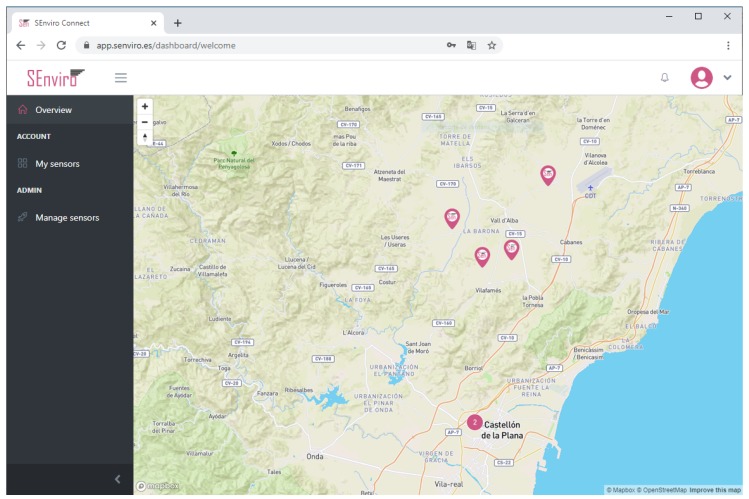
A screen capture of the *SEnviro* connect, where it shows all *SEnviro* nodes deployed.

**Figure 10:**
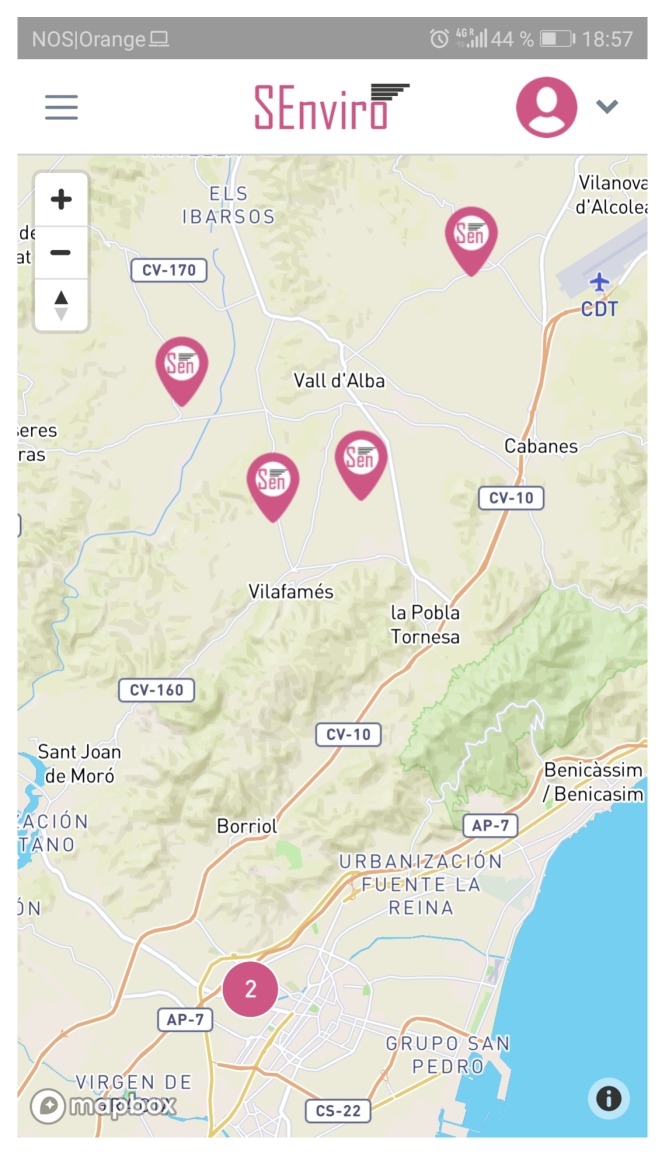
A screen capture of the *SEnviro* connect in a responsive design.

A web application using JavaScript, HTML5 and Cascading Style Sheets (CSS) technologies has been created (Figure 9). More concretely, Angular framework has been used to develop the web application following responsive design philosophy (Figure 10). the web app has different capabilities to fulfil regarding the *business* layer defined in the described IoT scenario. Two basic views have been designed: the first view displays the *SEnviro* node status (GPS position, last connection, battery, location and node alerts), whereas the second one is more focused on the vineyard cultivation perspective.

**Figure 11:**
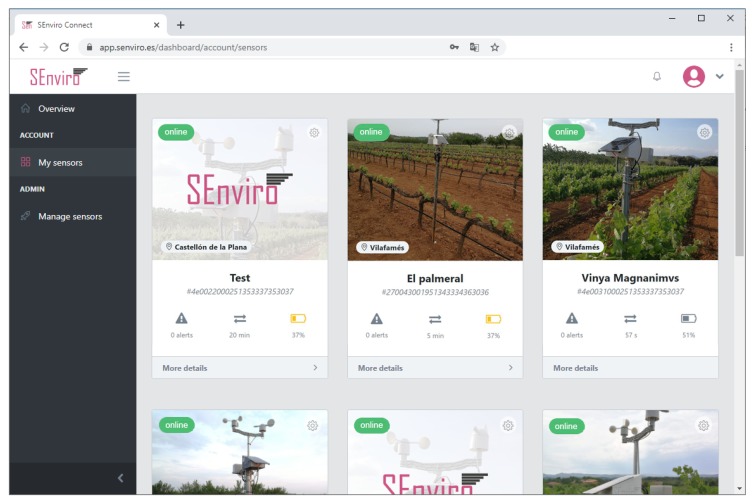
A screen capture of the *SEnviro* connect, where it shows the IoT device management view.

**Figure 12:**
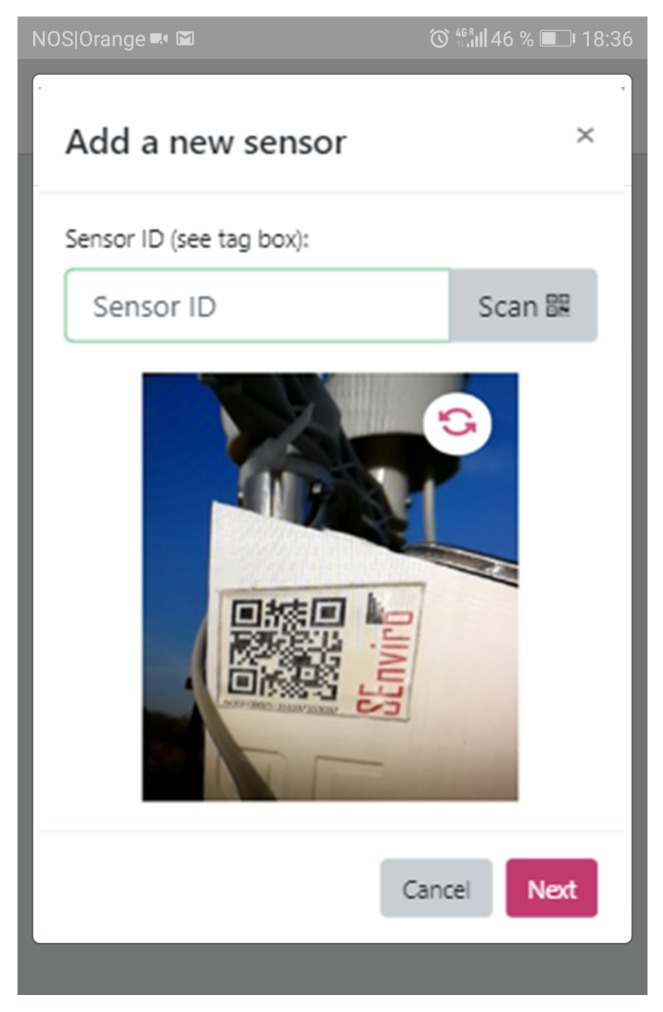
A screen capture of the *SEnviro* connect, where the wizard to add IoT devices is shown.

Figure 11 shows how we can handle the *SEnviro* nodes. New IoT devices can be added or updated using this view. Each *SEnviro* node holds a QR code, and the IoT device can be added by scanning its QR using the mobile camera or adding its ID (Figure 12). To provide information about the parcel where the IoT device is installed (GPS coordinates, holding name, and a photo of the IoT device deployment) a wizard has been developed to provide. When a new node is added, it is listed, and some information about it (battery percentage and status information) is shown.

**Figure 13:**
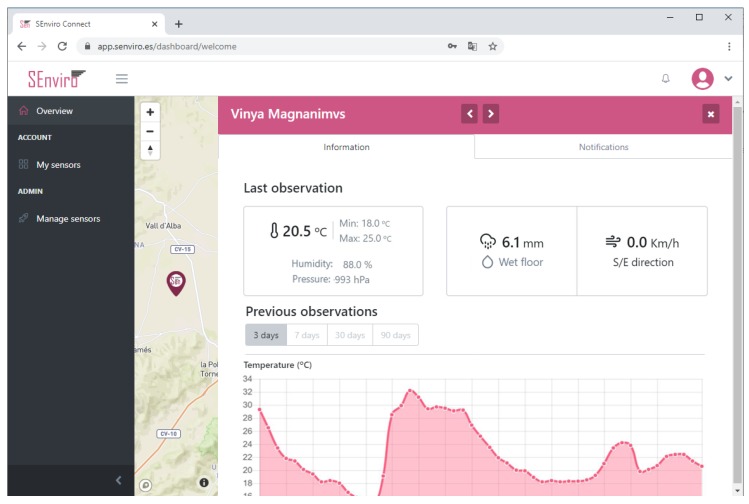
A screen capture of the *SEnviro* connect, showing the data and alerts for each IoT device.

The other view is designed to visualise observations and alerts in a manner which is adapted to the vineyard farmers (Figure 13). the *SEnviro* nodes are shown on a map using pins. Each pin opens a new lateral window to visualise each phenomenon observations in different line charts (adding different zooms). Finally, we can use this view to visualise the latest detected alerts from each supported disease model.

The client also provides administrator user access. This role has all privileges, and therefore can visualise all IoT devices and register new ones.

To develop the required web app, several frameworks have been used. In particular, we adopted a combination of already existing frameworks and libraries:

- Angular. An open source web application framework: https://angularjs.org(accessed on 20 March 2020). It provides a client-side framework for the Model-View-ViewModel (MVVM) architecture pattern [49]. It is combined with Bootstrap to offer a responsive solution.
- Mapbox. A JavaScript library that uses WebGL to render interactive maps from vector tiles and styles: https://www.mapbox.com/ (accessed on 20 March 2020).
- Chart.js. It is a library for integrating charts into modern web apps: https://www.chartjs.org/ (accessed on 20 March 2020).

To test the web app, four vineyard farmers have been involved in this project. These four *SEnviro* nodes were deployed in their respective land smallholdings. Some changes and design considerations relating to the visualisation of the information were added based on their opinions.

## 6. Performance Analysis: Scalability and Stability

This section covers the performance evaluation of the *SEnviro* connect following the previous approach and metrics introduced in [50]. To carry out this evaluation, a second computer has been used to generate random data and simulate IoT devices as publishers and users to retrieve observations using the web app developed. the aim of this experiment is to analyse the IoT platform in two aspects:

- Scalability. the IoT platform should support managing thousands of IoT devices publishing observations with a minimum delay to help time-critical applications. Throughput and response time metrics are valid indicators about how scalability is maintained. A high throughput rate means excellent performance, and it should scale linearly as several devices increase. Throughput is calculated as the number of messages received and processed by the IoT platform in a second. Low response time means fast response and processing of the platform.
- Stability. the stability of the system measured using several factors such as CPU and active memory usage. CPU usage is indicated by the average OS core utilisation of all CPU physical (and virtual) cores. Active memory displays the number of Megabytes (MB) stored in memory during the experiment by the time of the test.

### 6.1. Evaluation Approach and Tools

Performance experiment has been divided into two parts: one from the IoT devices perspective, and other from the user side as observations consumers. For the first case, we simulate 1 to 1000 virtual IoT devices or publishers distributed in 13 incremental steps (1, 2, 5, 7, 10, 25, 50, 75, 100, 250, 500, 750, 1000) using JMeter [51] with the MQTT plugin as a load generator. Virtual IoT devices send requests or messages with different rates, 10, 25, 50, 100 and 200 requests or messages per second, to the IoT platform. For this experimentation, only the MQTT publishing operation with QoS level 0 has been taken into consideration. the data used to make the ingestion has been simulated and represents an observation of a unique and random phenomenon; its average size is 46 bytes. the response from the server is always 20 bytes. During and after finishing our experiment, with the aim to collect CPU and memory usage metrics, we have used Prometheus [52] monitoring platform in combination with Google’s cAdvisor [53], a container resource usage analyser.

From the user’s perspective, we have made a similar but more restrained configuration. In this case, we have simulated the same amounts of users than IoT devices, but we have used different rates: 5, 10, 15 and 20. Requests for experimentation are made over two HTTP operations. In each iteration, the operation of returning the last observation of a random phenomenon (~179 bytes) and the operation of returning aggregated data are consulted. the latter returns a mean aggregate of seven days each two hours over a random phenomenon (~3000 bytes). the responses from both operations are ~179 and ~253 bytes, respectively. All requests are executed without caching responses between requests. As with the observation ingestion performance evaluation, the CPU and memory usage metrics have also been collected in this part.

Table 2 displays the specifications and environments of the computers used in the experiment.

### 6.2. Experiment Results

#### 6.2.1. Scalability

**Figure 14:**
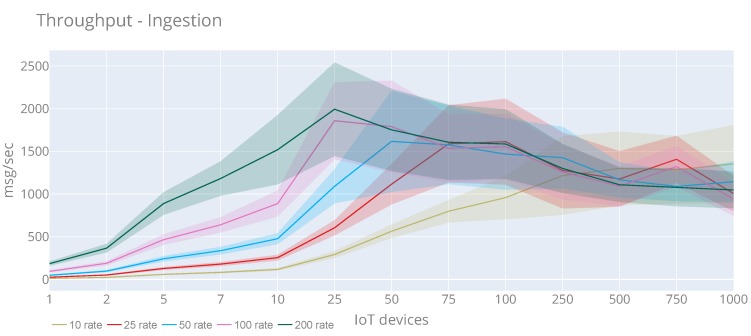
Average and standard deviation throughput of the observations injection with rates of 10, 25, 50, 100 and 200 requests per second.

Figure 14 displays the throughput results in case of IoT devices perspective performance evaluation. This chart shows the average using lines, and the colour areas represent the standard deviation (σ). Regarding throughput, we can see that the maximum value (2472 msg/sec) and maximum average (1994 msg/sec) are reached with 25 IoT devices and a rate of 200 msg/sec sent from each IoT device. From 25 IoT devices, the number of messages is decreased, and other rates follow this pattern. This is not due to lack of scalability of the IoT platform, because, as we will see in the CPU and RAM tests, they are not affected excessively. the reason is that the computer used to run the experiment cannot compete against the observations ingestion rate of the server. To further test from this point, more testing computers should be added to the evaluation. Authors consider that this is currently out of the scope of this paper, given that the presented results resemble sufficient to demonstrate the scalability of the IoT platform.

In total, 3,641,123 have been published, and only 112 timeout errors (~0%) have been obtained. the response time graph has not been included in this case, as, considering only the publication operations, the response time is linear, and does not depend on the number of IoT devices or rate.

**Figure 15:**
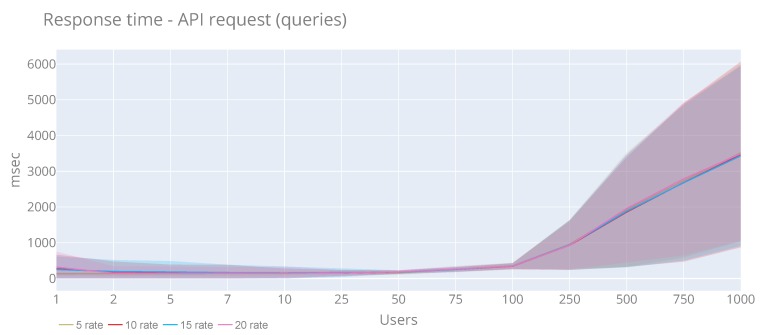
Average and standard deviation response time of API queries with a rate of 5, 10, 15 and 20 requests per second.

**Figure 16:**
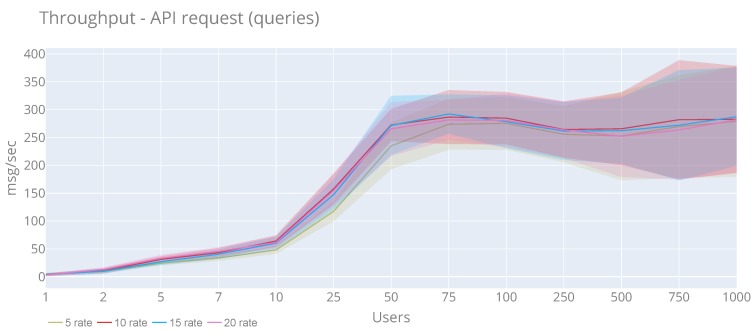
Average and standard deviation throughput of the API queries with a rate of 5, 10, 15 and 20 requests per second.

Figure 15 and Figure 16 display the results of the time response and throughput in case of users perspective. In both cases, we see that the rate is not affected by the performance. In the case of response time, for all rates, it is increased linearly starting from 100 users. the highest response time has been during 1000 users and rate 20, reaching 32,280 ms and an average of 3365 ms. Concerning the throughout, we see that all the rates follow the same pattern, in which from seven users, the time is increasing considerably. the maximum value (793 msg/sec) is reached with 1000 users and rate of 20. Moreover, the maximum average (292.22 msg/sec) is obtained with 75 users and rate of 15. In total, 525,792 have been published, and only 18,777 timeout errors (3.57%) have been obtained

#### 6.2.2. Stability

**Figure 17:**
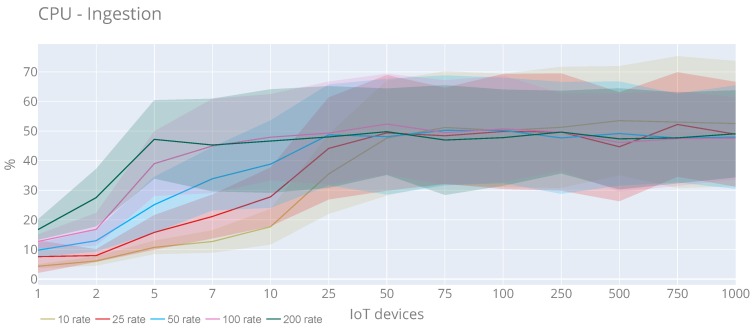
CPU utilisation of the observations injection with a rate of requests 10, 25, 50, 100 and 200 requests per second.

**Figure 18:**
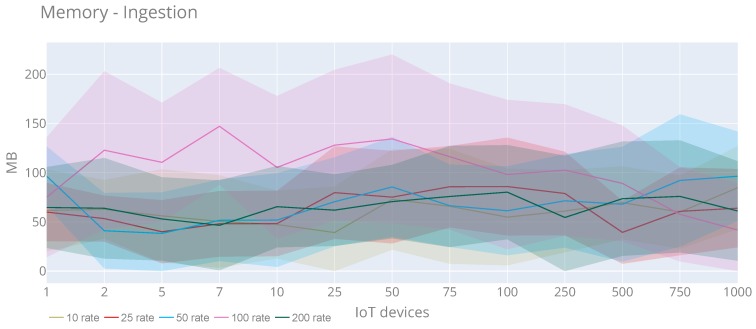
Memory usage of the observations injection with a rate of requests 10, 25, 50, 100 and 200 requests per second.

From the ingestion side, Figure 17 shows CPU usage at the platform’s evaluation time during all the experiment tests. We see that the pattern is similar for all rates, with a special mention to 200 rate test, when the CPU reaches the maximum speed. Although we can stress that it is not significant, the maximum value (70.62%) is reached with 750 IoT devices and rate 10. Using the same rate and with 500 IoT devices the maximum average of CPU usage (53.51%) is achieved. Concerning memory use (Figure 18), we see that there are no substantial changes when increasing IoT devices either and users. In this case, the maximum value (256.19 MB) is obtained with 50 IoT devices and rate 100. Moreover, the maximum average (147.12 MB) is reached with seven IoT devices and rate 100.

**Figure 19:**
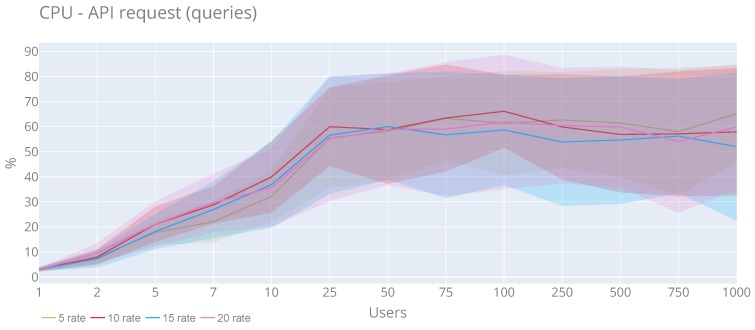
CPU utilisation of the API queries with a rate 5, 10, 15 and 20 requests per second.

**Figure 20:**
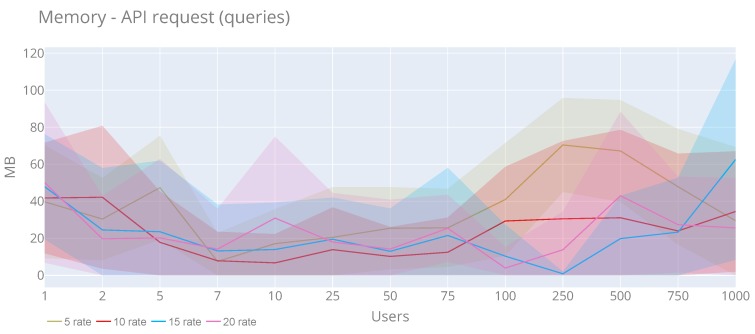
Memory usage of the API queries with a rate of 5, 10, 15 and 20 requests per second.

From the users’ perspective (Figure 19 and Figure 20), they show a behaviour similar to the previous graphs without any particularity. In the case of the CPU, the maximum value (81.39%) is obtained with 75 users and a rate of 20, and for the maximum average (0.66%) is reached with 100 users and rate 10. For memory (a peak of 116.72 MB) with 1000 users and a rate of 15 and the maximum memory average is 70.42 MB with 240 users and rate 5.

## 7. Related Work

In the market, there are several IoT platforms available in the form of pay-per-service cloud services (DeviceHive, Amazon AWS IoT or Azure IoT Suite, among others). One of the main features of the project is to use open source software. Some open source IoT platforms (Kaa, FIWARE or ThingSpeak) were evaluated (in 2017). Among them, the FIWARE platform was the most advanced at that time. Kaa and ThingSpeak were in their infancy and did not offer the variety of features they offer today. the decision to develop our own IoT platform was due to certain key functionalities of the project, such as a microservices architecture to guarantee service availability to our winegrowers during the development step and the ability to manage updates on IoT devices. There were not either powerful tools to facilitate the creation of custom clients for data visualisation. In addition, performance requirements were not considered.

Focused on research works from academia where they use non-generic and self-created IoT platforms, we have selected some approaches similar to our proposal. the following items provide an overview of some similar studies that have been identified and therefore analysed. the primary objective behind the analysis of these studies is to propose and validate an IoT platform architecture in an IoT scenario. Next, all works will be described.

- The authors of [54] present a conceptual and implemented IoT platform for gathering, analysing and sharing of groundwater data for different purposes.
- The authors of [55] propose the design of a smart IoT communication system manager used as a low-cost irrigation controller.
- In [56], the authors propose a platform that implements a microservice IoT-Big Data architecture supporting the distributed publication of multidisciplinary simulation models. the platform manages streams of data coming from the shop-floor in an optimised way for real-digital synchronisation, ensuring security and confidentiality of sensitive data.
- The study presented in [57] proposes an industrial IoT and communications at the edge framework, which has some stand-out features related to the easy integration of devices used in industrial environments with automatic configuration features, as well as the integration of multiple technologies.
- In [58], an IoT platform is proposed, which provides comprehensive support for multi-level provisioning of IoT Cloud systems. It provides and enables application-specific customisation of Edge devices. the IoT platform is evaluated using real-life applications in the domain of building management systems.
- In [59] propose an IoT platform called CHARIOT, which devises a runtime environment integrating heterogeneous resource-constrained IoT devices communicating with various protocols, as well as a scalable and dynamic communication layer that abstracts the connected devices and enables their intercommunication.
- The authors of MANDAVA et al. [60] present a distributed IoT platform infrastructure using machine learning computing technologies for air pollution monitoring in South African cities. the authors collect real-time environmental data, analyse the data collected from specific locations to determine whether the examined area is polluted or not and further compare performance measures of various machine learning algorithms adopted.

In order to compare the studies reviewed above, Table 3 presents a comparison between them. the following features to characterise each one have been proposed:

- Device Management: indicates if the IoT platform has the capability to manage devices. Scale: Yes/No.
- Interoperability: specifies if the IoT platform supports different technologies and protocols to connect IoT devices. Scale: Yes/No.
- Microservices: indicates if the system uses microservices. Scale: Yes/No.
- Serverless: shows if the system takes advantage of the serverless paradigm. Scale: Yes/No.
- IoT full stack: specifies if the study proposes a solution for all IoT parts. Scale: Yes/No.
- Alerts/Analysis: shows if the system applies some kind of analysis. Scale: Yes/No.
- Reusability: indicates if the system can be reused using container-based technologies [61]. Scale: Yes/No.
- Smart factor: the smart factor covered by each work. Scale: smart farming, smart environment, smart mobility, etc.

Table 3 shows that all studies provide a device management system to add/remove IoT devices. Although some works support a management devices capability, many do not offer an easy-to-use wizard for end-users for such *SEnviro* Connect proposes. Only three studies propose a system with the feature to connect IoT things using different protocols and technologies. For instance, the system in [58,59] supports MQTT and Constrained Application Protocol (COAP) connections. In our case, RabbitMQ provides additional protocols from those previously mentioned, such as STOMP and HTTP.

From a microservices point of view, only two studies [56,58] make use of this pattern. This kind of architecture seems too new, and so there are still few studies which make use of microservices.

Among the selected studies, none uses a serverless paradigm; only our study follows this approach. Serverless is one of the latest paradigms in computer science, so its usage has not yet been extended.

Some studies propose an IoT full-stack, among them, are Ciavotta et al. [56], Gaitan et al. [57], Nastic et al. [58], Akpolat et al. [59], MANDAVA et al. [60]. the studies listed recognise the use of IoT devices, but only our study incorporates its own IoT device design.

Finally, we can conclude that smart industry is the main smart factor where this kind of IoT platform is applied; this is because there is a significant interest in improving business within the industry and proposing systems that interconnect everything to everything in factories.

Among the related work included, no study adds a platform performance analysis. Neither of the works offers an agnostic vision of the platforms that enables their applicability in other different fields in which they are presented. Last, only one work [58] and our platform offer reusability using Docker as container-based technology.

## 8. Conclusions

In this work, we have presented a comprehensive solution to manage the whole IoT lifecycle. As has been presented, the lifecycle has four stages: Capture, Communicate, Analyse and Act (C2A2). A generic architecture based on these layers has been defined, which coincides with the stages of the IoT lifecycle. This architecture is granted to comply with the main requirements of IoT solutions.

IoT solutions have to deal with different challenges. One of the most critical of these is the heterogeneous nature of IoT devices. These devices have low computing capacity and energy restrictions. IoT devices are installed in hostile environments, and sometimes without access to electricity, they have to use batteries with energy-saving techniques. As indicated above, one of the most critical parts of the defined architecture is the IoT platform. the IoT platform must be able to solve all listed issues and requirements and abstract them for easy use by third parties. In what follows, the functional and non-functional (QoS) requirements of the proposed technological system are analysed.

At the functional level, the proposed IoT platform can **handle and discover IoT** for easy use and connection. To achieve that aim, some serverless functions are defined for devices and user management. Another requirement is **data management**. This is handled using Influxdb and Kapacitor; both tools are responsible for processing, storing and analysing the data gathered. Kapacitor supports an **event management** system. It offers a complete notification system and supports different ways to deliver notifications such as email, Slack and HTTP, among others.

Moving on to the non-functional level. Concerning **scalability**, the microservices architecture and the broker are used to guarantee scalability to add IoT devices. the comprehensive performance evaluation conducted over a single server ensures its scalability and stability in other complex deployments with more capacity. For example, 0% losses have been obtained in the ingestion system of the platform and throughput rates of more than 2400 msg/sec. In addition, the consumption at the CPU and memory levels (**stability**) are stable and contained throughout the test [62]. A container-based solution is proposed using Docker, and it offers satisfying horizontal scalability and **reusability**. Regarding **interoperability**, the broker (RabbitMQ) offers different protocols such as MQTT, COAP, HTTP and STOMP. the MQTT protocol provides sound energy efficiency in IoT devices [36]. the ingestion component guarantees the **real-time** feature through a microservice, with the added thing that Influx tools can process and analyse the data in real-time. The **reliability** of the system is also backed up due to RabbitMQ and Influx, as they are fault-tolerant tools. the **availability** of the system is partially guaranteed as part of the components follow serverless computing. Subtraction of services that do not use serverless functions can be derived to a BaaS to guarantee their availability. A TSL encryption using token-based authentication is offered through the architecture to guarantee the **security**. the web app also uses the same level of security. All components follow a container-based design through Docker to ensure ease of system deployment.

The IoT platform presented in this paper has been validated and used in a smart farming scenario. Two main components are presented: the IoT device called *SEnviro* node, and an IoT software platform, called *SEnviro* connect. Both parts work together to monitor vineyards, producing some advantages such as an autonomous (both in terms of energy and connectivity) IoT node; collecting meteorological conditions in vineyard fields; and following models to detect and alert diseases such as *Black rot*, *Downy mildew*, *Botrytis* and *Powdery mildew*. Other kinds of alerts, more focused on the IoT devices, which indicate the node state (online, battery, last connection) are supported. the validation was carried out over 140 days during a vineyard season (2018), and five *SEnviro* nodes were deployed. During this period, different alerts were detected that corresponded to the follow-up of the conducted crop models. Different farmers have used and validated the web application, contributing improvements in the initially proposed design and some of these improvements will be developed as future work.

In terms of costs, the IoT platform is relatively cheaper than other systems based on proprietary or as a service software, as it only requires the cost of a server and maintenance since it is built using open software. In addition, in the vineyard use case, a single implementation (a platform instance on a single server), as we have seen in the performance assessment, can support millions of IoT devices producing observations at the same instant. That implies thousands of farmers with various vineyard smallholdings can use the same server and save implementation costs (excluding the cost of the *SEnviro* nodes [33]).

Finally, in terms of future work, we are interested in conducting standards adoption to improve the IoT platform interoperability. For example, an adapter, following the approach in [63], could be developed to maintain OGC standards such as SensorThings API [64] and Sensor Observations Service (SOS) [65]. Other smart scenarios, such as smart health or smart cities, should also be planned in order to validate the IoT platform in different use cases. From the smart farming sphere, an in-depth validation of the launched alerts is proposed, in order to interpret success or failure in predictions. Currently, only a standalone node strategy is supported, and we consider that a policy where several IoT devices are involved would cover a smallholding with higher precision. Interpolation techniques from different IoT devices data could be supported to cover entire fields and vast areas, this information could be combined with remote sensing data such as Copernicus satellites. Another possible extension of the IoT platform is to add features to support IoT devices mobility [66].

## Figures and Tables

**Table 1:** Locations of *SEnviro* nodes used in this project.

*SEnviro* Number	Location (x, y)
1	39.993934, −0.073863
2 (Figure 8)	40.133098, −0.061000
3	40.206870, 0.015536
4	40.141384, −0.026397
5	40.167529, −0.097165

**Table 2:** Specifications of two environments used in this performance analysis.

Specification	Test Runner	Deployment Server
CPU	8x Intel(R) Core i7-3770K@4.30 GHz	4x Intel(R) Xeon(R) CPU E5-2690 v2@3.00 GHz
Memory	16,384 MB	16,384 MB
OS	Windows 10	Ubuntu 16.04.4 LTS
Installed software	JMeter 5.2.1	PostgreSQL 10.3, Micro services (measurement, ingestion, device), chronograf 1.4.4, Kapacitor 1.4.1, InfluxDB 1.5.1, Consul, RabbitMQ, Prometheus server and cAdvisor

**Table 3:** Comparison between different related studies.

Work Reference	Device Management	Interoperability	Microservices	Serverless	IoT Full Stack	Alerts/Analysis	Reusability	Smart Factor
[54]	✓	✗	✗	✗	✗	✓	✗	Cities
[55]	✓	✓	✗	✗	✗	✓	✗	Farming
[56]	✓	✗	✓	✗	✓	✓	✗	Industry
[57]	✓	✗	✗	✗	✓	✓	✗	Industry
[58]	✓	✓	✓	✗	✓	✓	✓	Environment
[59]	✓	✓	✗	✗	✓	✓	✗	Industry
[60]	✓	✗	✗	✗	✓	✓	✗	Environment
Ours	✓	✓	✓	✓	✓	✓	✓	Farming
